# Pandora's Box

**DOI:** 10.1192/bji.2022.33

**Published:** 2023-02

## Cannabis, pregnancy and children

Cannabis has been in the limelight for some time for a variety of reasons, including its medicinal benefits and easing of restrictions on its use in many parts of the world. Public attitudes have relaxed as more powerful drugs attract more attention to their risks. Medicinal use may have influenced public thinking and possible lack of understanding of the versions of cannabis available and the relevance of the THC (delta-9-tetrahydrocannabinol) component.

In the USA, there has been a dramatic increase in the use of cannabis during pregnancy, which has stimulated interest in examining the possible effects on the offspring of user mothers. Non-clinical evidence shows that THC crosses the placenta and may potentially affect the brain development of the offspring.

Researchers had previously found that maternal cannabis use during pregnancy was associated with increased psychopathology in middle childhood. In a recently published study, following up the original data from the ABCD (Adolescent Brain Cognitive Development) study, they examined the possibility that psychopathology persists into early adolescence.

Following the STROBE (Strengthening the Reporting of Observational Studies in Epidemiology) guidelines for cohort studies, they used mixed models to estimated associations between retrospective reports of cannabis use in three maternal groups (group 1, cannabis use during pregnancy, before mothers became aware of their pregnancy; group 2, cannabis use both before and after knowledge of pregnancy; and group 3, no exposure to cannabis) and child psychopathology.

A total of 10 631 mothers participated in the study, with the following numbers in each group: group 1, *n* = 391; group 2, n = 208; and group 3, *n* = 10 032. Of these, 81% were White, 22% were African American, 7% were Asian or Asian American, 7% were Hispanic, 4% were native American and 7% were other. The researchers assessed children for psychopathology, at baseline and at follow-up after 1 and after 2 years. Cannabis exposure during pregnancy was associated with persisting psychopathology (attention, social and behavioural problems) throughout early adolescence and did not change with age. This raises serious concerns about the effects of the substance on neurodevelopment, which may increase vulnerability in these children to later mental disorders and substance misuse.

Pregnant women are advised against alcohol use, drug use and cigarette smoking during pregnancy. This study highlights the importance of more specific advice on the detrimental effects of cannabis on children.

Baranger DAA, Paul SE, Colbert SMC, Karcher NR, Johnson EC, Hatoum AS,  Association of mental health burden with prenatal cannabis exposure from childhood to early adolescence: longitudinal findings from the Adolescent Brain Cognitive Development (ABCD) study. JAMA Pediatr 2022; 176(12): 1261–5.3609459910.1001/jamapediatrics.2022.3191PMC9468940

## Of children and sleep

It is suggested by the American Academy of Sleep Medicine that 6–12-year-olds should sleep for 9 h per day. As it is known that children often sleep less than this, a recent study aimed to examine whether and how insufficient sleep may affect their mental health, cognition, and brain function and structure during this crucial period of neurocognitive development.

They carried out a longitudinal, observational cohort study using data from the ABCD study. The participants were divided into those with sufficient and those with insufficient sleep (cut off point of 9 h) and were matched on 11 covariates including gender, family income, body mass index and puberty status. The researchers obtained ABCD data on behavioural problems and mental health, cognition, and structural as well as resting-state functional brain measures, as assessed at baseline and at 2 years. Over 8000 children were included in the study, with 3021 matched pairs (sufficient versus insufficient sleep) at baseline and 749 pairs at 2 years. There were similar between-group differences at baseline, which persisted at 2 years, in both behavioural measures and structural and functional brain measures. Children with insufficient sleep had more mental and behavioural problems (impulsivity, stress, depression, anxiety, aggressive behaviour and thinking problems) compared with those with sufficient sleep. They also had impaired cognitive function (decision-making, conflict-solving, working memory and learning). There were also structural and functional brain differences between the two groups. Children with insufficient sleep had reduced thickness of grey matter or smaller volume in certain areas of the brain that are responsible for attention, memory and inhibition control.

The importance of adequate sleep in young children and in particular during the adolescent period when neurodevelopment occurs cannot be overestimated.

Yang FN, Xie W, Wang Z. Effects of sleep duration on neurocognitive development in early adolescents in the USA: a propensity score matched, longitudinal, observational study. Lancet Child Adolesc Health 2022; 6(10): 705–12.3591453710.1016/S2352-4642(22)00188-2PMC9482948

## Memories

How do we keep our memories alive and not interfered with by emerging new memories? How does our brain consolidate our memories, good or bad? We know that the hippocampus is a key area of the brain in the process of memory-storing, and we also know of the importance of healthy neurons and neuroplasticity, but how are our memories stabilised in our brains and remain clear and distinct?

Researchers from Bristol University, UK, working with computational neuroscientists at Imperial College London, claim to have worked out the mechanism and the systems involved in this process. Strengthening the connections between neurons ensures the creation of memories. More specifically, the researchers claim a strong involvement of inhibitory patterns of interneurons, in addition to excitatory signalling. They demonstrated that inhibitory synapses from parvalbumin-expressing interneurons undergo long-term depression, whereas somatostatin-expressing interneurons undergo potentiation, during physiological activity. These interneurons target the dendrites of CA1 pyramidal cells, which coordinate excitatory inputs from the entorhinal cortex and CA3.

Using an optogenetic approach to hippocampal slices, selectively activating dendrite inhibition of CA1 neurons, they showed that these forms of long-term inhibitory plasticity have significant effects on the output of CA1 pyramidal neurons. By means of computational modelling, they demonstrated that this inhibitory activity enables the hippocampus to stabilise any changes to excitatory strength, which prevents new information from interfering with established memories.

This process enables us humans to retain memories and expectations and make reasonable predictions about the future.

Udakis M, Pedrosa V, Chamberlain SEL, Clopath C, Mellor JR. Interneuron-specific plasticity at parvalbumin and somatostatin inhibitory synapses onto CA1 pyramidal neurons shapes hippocampal output. Nat Commun 2020; 11: 4395.3287932210.1038/s41467-020-18074-8PMC7467931

### Of men and women – the ‘Matilda effect’

In the modern world of the 21st century, we are progressively closing the gender gap in many professional spheres, and women are encouraged to follow a career in science. However, is this just a ‘politically correct’ approach without substance, or are women indeed valued as much as their male colleagues? Alas, the evidence shows otherwise. Women's contribution to scientific scholarship is not acknowledged enough, as shown by the citation gap between men and women authors (the ‘Matilda effect’). Research findings point to differences in scientific attribution rather than contribution of women scientists as responsible for the observed gender gap. Women are less likely than men to be credited with authorship, and this is apparent in different sources of data including large administrative data on research teams, team scientific output and attribution of credit, surveys of authors and qualitative responses. This gender gap is apparent in many scientific fields and at many career stages.

Women researchers are less likely to be cited than their male peers. Over-selection of men and under-selection of women of similar quality were demonstrated in a recent study. A further finding was a ‘first mover advantage’, which demonstrated that among papers of similar type, those authored by men were likely to be published before those authored by women. This offers men an unfair advantage to progress in their careers earlier than women.

Experts have considered ways of achieving gender parity, and the Gender Balance Assessment Tool was produced as a strategy to quantify the proportions of papers authored by women and men and provide information on the citation parity in studies. This is claimed to also signal a commitment to diversity, equity and inclusion. The authors stress the need for collective involvement, including individual researchers as well as journals and academic institutions, in order to achieve parity.

Ross MB, Glennon BM, Murciano-Goroff R, Berkes EG, Weinberg BA, Lane JI. Women are credited less in science than men. Nature 2022; 608: 135–45.3573223810.1038/s41586-022-04966-wPMC9352587

Teich EG, Kim JZ, Lynn CW, Simon SC, Klishin AA, Szymula KP,  Citation inequity and gendered citation practices in contemporary physics. Nat. Phys. 2022; 18: 1161–70.

### Celebrity status – the ‘Mathew effect’

In 1968, sociologists Robert Merton and Harriet Zuckerman coined the term ‘Mathew effect’ to describe how high-status researchers ‘tend to get disproportionately more of the same’. A group from the University of Innsbruck decided to check on the current validity of this concept. They contacted by email 3300 researchers asking them to review an economics study for publication in a journal. The proposed publication was authored by Vernon Smith, a past Nobel laureate in economics with 54 000 citations and one of his former PhD students, Sabiou Inoua, with only 42 citations on Google Scholar. The peer reviewers were sent three different versions of the paper, with one listing only V. Smith as the author, a second one listing only Inoua and the third one without an author name.

A total of 821 researchers agreed to review the paper; 38.5% of those who were given Smith's name agreed to go ahead with the review, compared with 30.7% of those given no name and 28.5% of those given Inoua's name. In a further check to avoid bias, one of three papers (listing only Smith, only Inoua or no author) was randomly assigned to those peer reviewers who had previously been given the paper with no author's name. They were also told that this was an experiment that included more than a few peer reviews (instead of the usual 2–3). It is no surprise that the paper listing Smith got the highest marks from the reviewers, who were also complimentary of him for including new information and conclusions supported by data. Twice as many (24%) accepted the no-author version for publication as those who were given the paper listing Inoua as the author. The possibility that a racial bias may have also played a role was noted, as Smith's name was clearly American and that of Inoua (a citizen of Niger and dark skinned) was not.

Juergen H, Inoua MS, Kerschbamer R, König-Kersting C, Palan S, Smith VL. Nobel and novice: author prominence affects peer review. Proc Natl Acad Sci USA 2022; 119(41): e2205779119.3619463310.1073/pnas.2205779119PMC9564227

## Parasites – friends or foes?

We know that some bacteria are good for us, and a healthy intestinal microbiome has beneficial effects on our physical and mental health. Interestingly, more complex organisms such as parasites may have behavioural effects, at least in wolves, according to a recent study published in *Nature*. It was observed (although the evidence was not consistent) that rodents infected with *Toxoplasma gondii* are less fearful of cats and that infected hyenas became more likely to be eaten by lions in Kenya.

Researchers from the University of Montana decided to examine the effects of the parasite on local wolves, who are known to be in contact with cougars, which carry *T. gondii*. After the acute infection of the animal, the parasites form cysts in muscle tissue as well as in the brain, where they persist for the rest of the animal's life. Does this affect the health of the host animal? This was the question the researchers aimed to examine.

They examined 256 samples from 229 wolves, which they had previously observed throughout their lives and for which they had details of their life histories and social status. To their surprise, they found that the infected wolves were 11 times more likely to leave their family group and start a new pack and 46 times more likely to actually lead the pack. The effect on the ecosystem is significant, and the authors conclude that ‘parasites might have a much larger role than anyone gives them credit for’. No evidence for such effects exists in humans (one-third of humans may also be chronically infected), in whom *T. gondii* infection has detrimental effects on health and is best to stay clear of!

Marris E. Parasite gives wolves what it takes to be pack leaders. Nature 2022; 612(7939): 202.3642450310.1038/d41586-022-03836-9

